# A novel, pathogenic dinucleotide deletion in the mitochondrial *MT-TY* gene causing myasthenia-like features

**DOI:** 10.1016/j.nmd.2020.06.008

**Published:** 2020-08

**Authors:** Albert Z. Lim, Grace McMacken, Francesca Rastelli, Monika Oláhová, Karen Baty, Sila Hopton, Gavin Falkous, Ana Töpf, Hanns Lochmüller, Chiara Marini-Bettolo, Robert McFarland, Robert W. Taylor

**Affiliations:** a Wellcome Centre for Mitochondrial Research, The Medical School, Newcastle University, Newcastle upon Tyne, UK; b The John Walton Muscular Dystrophy Research Centre, Newcastle University, Newcastle upon Tyne, UK; c NHS Highly Specialised Service for Rare Mitochondrial Disorders, Newcastle upon Tyne, UK; d Department of Neurosciences, Royal Victoria Hospital, Belfast Health and Social Care Trust, Belfast, UK; e Biosciences Institute, Faculty of Medical Sciences, Newcastle University, Newcastle upon Tyne, UK; f Department of Neuropediatrics and Muscle Disorders, Medical Center, Faculty of Medicine, University of Freiburg, Freiburg, Germany; g Centro Nacional de Análisis Genómico (CNAG-CRG), Center for Genomic Regulation, Barcelona Institute of Science and Technology (BIST), Barcelona, Spain; h Research Institute, Children's Hospital of Eastern Ontario, Ottawa, Canada; i Division of Neurology, Department of Medicine, Ottawa University, Ottawa, Canada; j Translational and Clinical Research Institute, Faculty of Medical Sciences, Newcastle University, Newcastle upon Tyne, United Kingdom

**Keywords:** Mitochondrial disease, Congenital myasthenia syndromes (CMS), *MTTY* gene, mtDNA tRNA variant, Muscle biopsy

## Abstract

Mitochondrial DNA (mtDNA)-related diseases often pose a diagnostic challenge and require rigorous clinical and laboratory investigation. Pathogenic variants in the mitochondrial tRNA gene *MT-TY*, which encodes the tRNA^Tyr^, are a rare cause of mitochondrial disease. Here we describe a novel m.5860delTA anticodon variant in the *MT-TY* gene in a patient who initially presented with features akin to a childhood onset myasthenic syndrome. Using histochemical, immunohistochemical and protein studies we demonstrate that this mutation leads to severe biochemical defects of mitochondrial translation, which is reflected in the early onset and progressive phenotype. This case highlights the clinical overlap between mtDNA-related diseases and other neuromuscular disorders, and demonstrates the potential pitfalls in analysis of next generation sequencing results, given whole exome sequencing of a blood DNA sample failed to make a genetics diagnosis. Muscle biopsy remains an important requirement in the diagnosis of mitochondrial disease and in establishing the pathogenicity of novel mtDNA variants.

## Highlights

• This novel m.5860delTA variant in the *MT-TY* gene that caused mitochondrial disorder can manifest clinical features suggestive of myasthenic syndromes.
• This variant has been demonstrated to be pathogenic by our histochemical, immunohistochemical and protein studies.
• Muscle biopsy remains an important diagnostic investigation in the era of next generation sequencing.

## 1 Introduction

Mitochondrial DNA (mtDNA) contains 13 genes that encode essential subunits of the oxidative phosphorylation (OXPHOS) complexes, 2 rRNAs and 22 tRNAs [1]. The mitochondrial tRNA (mt-tRNA) genes, which contribute less than 10% of the total coding sequence of the mitochondrial genome, are known as pathogenic ‘hotspots’ because they are responsible for more than half of the mtDNA-related diseases [2,3]. The point mutations in these mt-tRNA genes typically cause a loss of its stability leading to defective mitochondrial translation and combined respiratory chain deficiency [4]. Affected individuals harbouring the same point mutation may have different phenotypes and conversely, identical mitochondrial syndromes can be caused by different genetic variants. This loose genotype-phenotype correlation in mtDNA-related disease can lead to diagnostic difficulties. Other neuromuscular disorders, such as myasthenic syndromes can often be mistaken for mitochondrial myopathies [5], [6], [7] and equally, diagnostic investigation of myasthenia syndromes should consider the possibility of mitochondrial disease. Therefore, a ‘novel’ mt-tRNA variant in the context of indistinct neuromuscular presentations requires rigorous clinical and laboratory evaluation to substantiate its pathogenicity [8]. To date, only a small number of mutations in the *MT-TY* gene (coding for mitochondrial tRNA Tyrosine) have been reported in the literature [9], [10], [11], [12], [13], [14]. We have identified a novel m.5860delTA variant in the *MT-TY* gene and confirmed its pathogenicity through detailed phenotyping in conjunction with functional laboratory work. This novel variant is the first dinucleotide deletion in the *MT-TY* gene, allowing us to directly compare its clinical and biochemical consequences with those of other pathogenic *MT-TY* gene variants.

## 2 Material and methods

### 2.1 Patient and clinical investigations

The patient was referred to the UK NHS Highly Specialised Services for Rare Neuromuscular Disease and Rare Mitochondrial Disease in Newcastle upon Tyne, UK. Informed ethical consent was obtained in accordance with the Declaration of Helsinki to publish relevant clinical material including photography. All clinical tests had been analysed in the Newcastle-upon-Tyne Hospitals NHS Foundation Trust. Open biopsy of the quadriceps muscle was performed electively under general anaesthetic.

### 2.2 Histopathological analyses

Standard histological and histochemical analyses were performed on fresh-frozen sections (10 µm) of skeletal muscle biopsy according to established protocols [15]. This included the assay of cytochrome *c* oxidase (COX) both individually and using a sequential COX/succinate dehydrogenase (SDH) assay. Additionally, mitochondrial OXPHOS function was assessed using a quadruple immunohistochemical assay of complex I (NDUFB8), complex IV (COX1) and porin (mitochondrial mass marker) immunoreactivity as previously reported [16].

### 2.3 Molecular genetic analyses

Total DNA was extracted from patient tissues and maternal samples by standard procedures. Exome capture of total blood DNA was undertaken using the Agilent SureSelect Human All Exome v5 kit and sequenced on an Illumina HiSeq4000 at the Centro Nacional de Análisis Genómico, Barcelona. Alignment and variant calling were carried out using Burrows–Wheeler Aligner and Genome Analysis ToolKit, respectively. VCF files were uploaded to the RD-Connect Genome-Phenome Analysis Platform (GPAP, https://platform.rd-connect.eu), for variant prioritization. The entire mitochondrial genome was amplified in two overlapping fragments by long-range PCR, and analysed by Next Generation Sequencing using an Ion Torrent™ Personal Genome Machine (PGM) platform (Thermo Fisher Scientific). Sequences were aligned to the revised Cambridge reference sequence (GenBank reference accession number: NC_012920.1) for human mtDNA [17]. Data analysis was performed in Torrent Suite v5.0.4 using Variant Caller v5.0.4.0 and Coverage Analysis v5.0.4.0.

### 2.4 Assessment of mutation load by quantitative pyrosequencing

Individual COX-positive and COX-deficient fibres were isolated by laser microdissection and lysed to obtain total DNA to be used in single-fibre mutation segregation studies. The mtDNA mutation load in homogenate tissues and individual COX-positive and COX-deficient fibres was determined by quantitative pyrosequencing, using mutation-specific primers (details available on request). Pyrosequencing was performed on the Pyromark Q24 platform according to manufacturer's protocol. mtDNA heteroplasmy levels were quantified by comparing peak heights of wild-type and mutant nucleotides at the relevant position.

### 2.5 Western blotting and Blue-Native page analysis

Sodium dodecyl sulphate (SDS) and one-dimensional Blue-Native (BN) polyacrylamide gel electrophoresis (PAGE) analysis was performed as described previously [18]. Briefly, skeletal muscle tissues were homogenised, pelleted and solubilised [19]. The supernatant was retained for SDS and BN PAGE analysis. Protein concentrations were determined using Pierce BCA Protein Assay kit. OXPHOS proteins and complexes were separated electrophoretically, transferred to PVDF membranes (Immobilon™-P, Millipore Corporation) and immunodecorated using OXPHOS-subunit specific primary antibodies and species-appropriate secondary antibodies. The following primary antibodies were used in this study NDUFB8 (Abcam, ab110242), SDHA (Abcam, ab14715), UQCRC2 (Abcam, ab14745), COX1 (Abcam, ab14705), ATP5A (Abcam, ab14748), total OXPHOS human WB antibody cocktail (ab110411) and MT-ND2. Chemiluminescence signal was detected using ECL prime kit (GE Healthcare) and ChemiDocMP Imaging System (Bio-Rad).

## 3 Results

### 3.1 Clinical description

A Caucasian male, born to non-consanguineous parents, had unremarkable birth history and normal early neurodevelopment in his first two years. His parents noticed delayed speech at age 4 years. At age 6 years, he developed ptosis of his left eye with subsequent progression over 12 months to bilateral ptosis and associated diplopia. He also experienced increasing difficulty with chewing and swallowing, resulting in weight loss. Compared to his peers, he was easily fatigued, typically towards end of the day and found physical activities more challenging. At age 8 years a diagnosis of congenital myasthenic syndrome (CMS) was considered, but trials of both pyridostigmine and oral salbutamol failed to achieve symptomatic improvement. Although his proximal muscle weakness and exercise intolerance fluctuated depending on the degree of exertion, he continued to slowly deteriorate. By age 12, he required a wheelchair for distances over 500 metres. Bulbar dysfunction also progressed, adversely affecting his nutrition and at the age of 13 years he required a percutaneous endoscopic gastrostomy (PEG). He had learning difficulties and required one-to-one support in school. There was no relevant family history of inherited conditions.

**Fig. 1:**
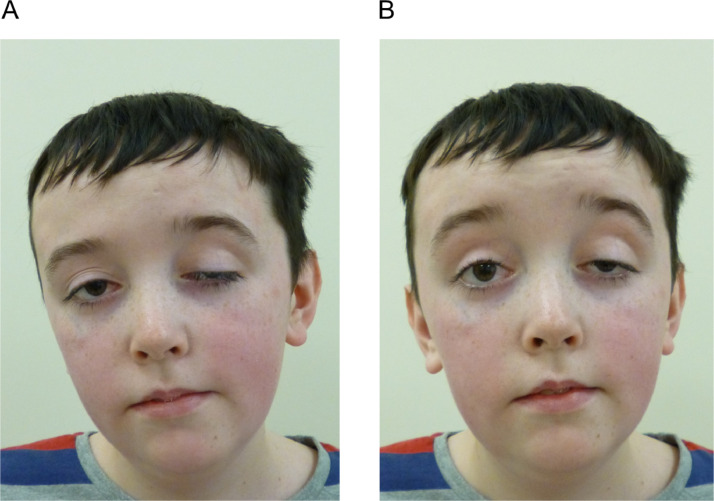
**Clinical photography of the proband displaying severe bilateral ptosis which was asymmetric.** (A) Symmetrical facial weakness and compensatory head tilt due to external ophthalmoplegia; (B) Ptosis is uncorrected with frontalis over-activation.

On examination at age 15 years, weight and height were on the 25th centile; a significant improvement from the 0.4th centile prior to PEG insertion. He had asymmetric ptosis (left worse than right), covering more than two thirds of his pupil with bilateral external ophthalmoplegia (Fig. 1). Neurological examination revealed periorbital and perioral weakness, with hypotonia and weakness of the neck, hip flexors and shoulder girdle (MRC 3+/5). He exhibited a positive Gower's manoeuvre and a myopathic gait.

Forced vital capacity was only 52% predicted. His polysomnography indicated borderline nocturnal hypoventilation although he had no morning headache or daytime sleepiness. He did not fulfil the criteria for overnight respiratory support but had to undergo 6-monthly follow-up with the respiratory team. Magnetic resonance imaging of head did not reveal any intracranial abnormality, and electrocardiogram and transthoracic echocardiography were normal. AChR and MuSK antibodies were negative and serum creatine kinase was normal. He had normal nerve conduction studies and baseline compound muscle action potential (CMAP) amplitudes. Although repetitive nerve stimulation revealed no significant increment or decrement at 3 Hz stimulation, single fibre electromyography (SFEMG) of extensor digitorum communis revealed increased jitter suggestive of a defect in neuromuscular transmission. His hearing and visual assessments were within normal limits for his age.

### 3.2 Nuclear genetic analyses

Given the initial presentation of predominant ocular and bulbar weakness, primary genetic testing was of the CMS related genes *CHRNE, CHRNA1, CHRNB1, COLQ, GFPT1* and *DPAGT1*. However, these were negative. Thus, the DNA from the proband was put forward to next generation whole exome sequencing (WES) using standard filtering criteria for rare diseases – minor allele frequency (MAF) <0.01, variant effect predictor (VEP) = mod/high, combined annotation dependent depletion (CADD) >20, a gene list comprising 416 genes known to be associated with neuromuscular disease and a recessive mode of inheritance (both homozygous and compound heterozygous). This filtering strategy however, failed to identify clear candidate variants. Relaxing the filters to also include variants with CADD scores of <20 and low VEP identified two *TTN* variants (ENSG00000155657) a predicted benign missense change (p.Thr21008Ile) and a synonymous change (c.14424G>C; p.Val4808), and two *NEB* variants (ENSG00000183091) an extended splice site (c.24208–7C>T) variant and a very rare, synonymous change (c.22776A>G; p.Lys7592) which was predicted to affect splicing (Human Splicing Finder, Version 3.1 (http://www.umd.be/HSF/). Of these, the findings of two heterozygous *NEB* variants prompted the muscle biopsy to demonstrate changes related to nemaline myopathy as a possible diagnosis.

### 3.3 Histopathological analyses of skeletal muscle

**Fig. 2:**
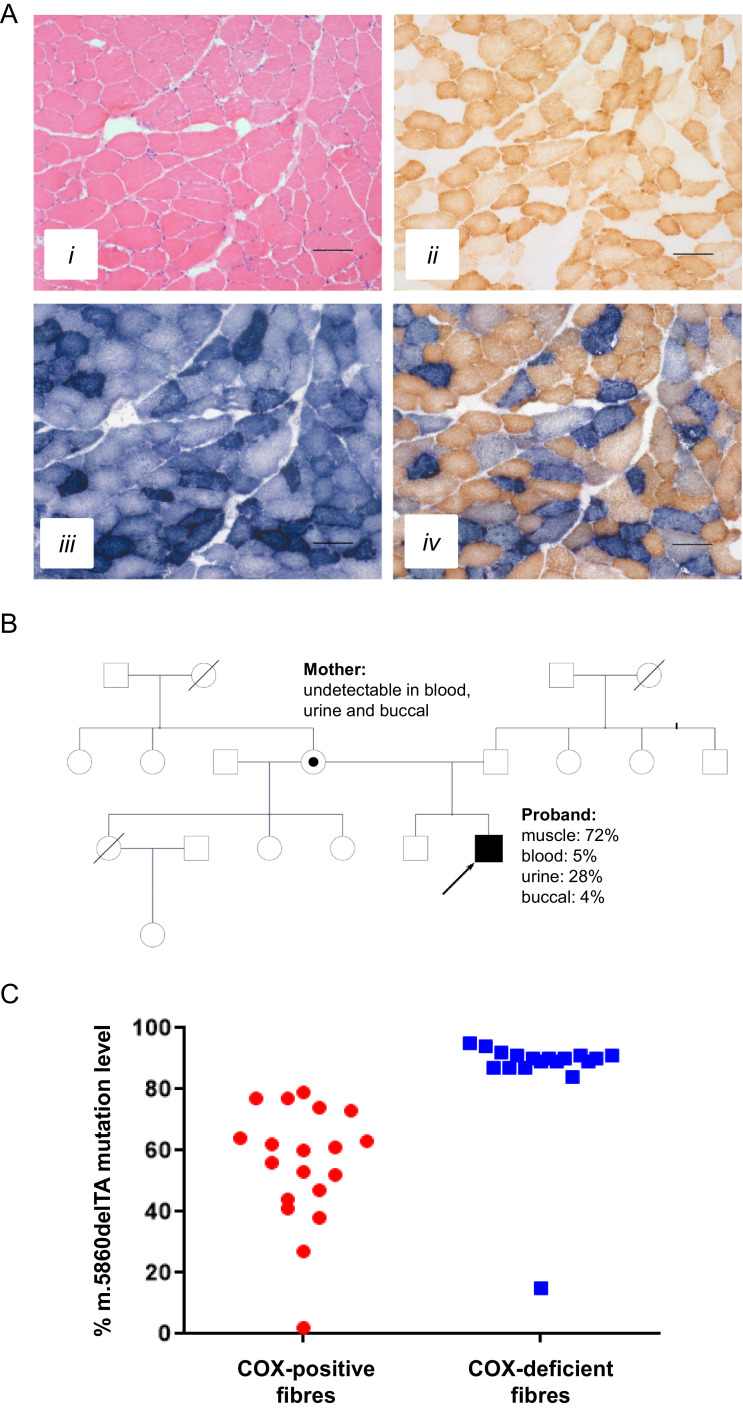
**Histopathological and molecular genetic studies.** (**A**) Hematoxylin and eosin (H&E) staining (*i*), cytochrome *c* oxidase (COX) histochemistry (*ii*), succinate dehydrogenase (SDH) histochemistry (*iii*) and sequential COX-SDH histochemistry (*iv*) demonstrate a clear mosaic pattern of COX deficiency with many fibres showing abnormal, subsarcolemmal accumulation of mitochondria. Scale bar = 100 µm. (**B**) Family pedigree showing the segregation of the novel *MT-TY* variant in different tissues in the patient, and absence of this variant in DNA samples from his mother. (**C**) Quantitative assessment of m.5860delTA mutation load in individual COX-deficient and COX-positive muscle fibres, confirming segregation of high mutant load with a biochemical defect.

Muscle biopsy from quadriceps femoris did not reveal any abnormalities in-keeping with a diagnosis of nemaline myopathy; there were no distinct rods or rod-like structures to support this candidate diagnosis. The histopathological analysis, specifically the oxidative enzyme histochemistry, did however, show a significant number of COX-deficient muscle fibres which were SDH-positive, affecting ∼25–30% of the total number of fibres in the biopsy. Approximately 10% of all fibres showed abnormal subsarcolemmal mitochondrial accumulation (ragged-red fibres) (Fig. 2**A**). These findings raised the possibility of a mitochondrial myopathy, and a deep analysis of the mitochondrial genome was initiated.

### 3.4 Mitochondrial genetic analysis

Large-scale mtDNA rearrangements were excluded in muscle using validated long-range PCR methods [20]. Subsequently, full sequencing of muscle mtDNA (haplogroup J1c) revealed a novel m.5860delTA *MT-TY* (mt-tRNA^Tyr^) gene variant which is predicted to result in the deletion of two nucleotides within the anticodon of the mt-tRNA^Tyr^ molecule. This is highly likely to result in an mtDNA translation defect due to mispairing with the corresponding mt-mRNA and thus result in a combined OXPHOS defect. The novel *MT-TY* variant was present at high levels of mtDNA heteroplasmy (72%) in the patient's skeletal muscle based on Ion Torrent NGS data. Pyrosequencing assays additionally quantified the m.5860delTA variant at low heteroplasmy levels in blood (5%), buccal (4%), and urine (28%) derived DNA samples; a pattern of variable segregation commonly observed for pathogenic mtDNA variants. The m.5860delTA mutation was not detected in non-invasively obtained DNA samples (blood, urine and buccal) from the patient's mother, suggesting that the mutation had arisen *de novo* (Fig. 2**B**). In addition, a retrospective review of WES data failed to identify the mtDNA mutation. This is because NGS was performed using a standard exome sequencing kit without additional mtDNA probes, meaning mtDNA mutations would not have been detected.

### 3.5 Single muscle fibre studies

Single fibres studies of COX-deficient and COX-positive fibres demonstrated the segregation of the m.5860delTA variant with the COX-deficient fibres in skeletal muscle (mutation loads: COX-deficient fibres 85.61 ± 4.20%, COX-positive fibres 55.26 ± 4.45%, unpaired T-test *P*=<0.0001), confirming pathogenicity of this novel mutation (Fig. 2**C**).

### 3.6 Immunoanalysis of mitochondrial OXPHOS subunits and complexes

**Fig. 3:**
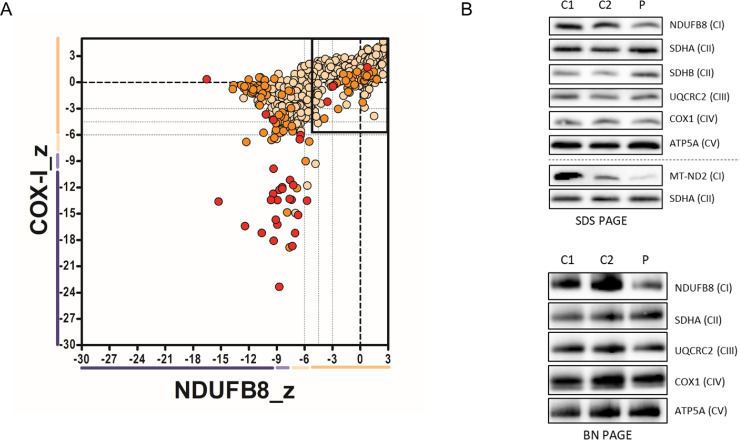
**Assessment of mitochondrial protein levels and OXPHOS complex assembly in patient muscle.** (**A**) Quadruple immunofluorescence analysis of NDUFB8 (complex I) and COXI (Complex IV). Each dot represents the measurement from an individual muscle fibre, colour co-ordinated according to its mitochondrial mass (normal = beige, high = orange, very high = red), grey dashed lines represent SD limits for classification of the fibres. Lines next to x- and y- axis represent the levels of NDUFB8 and COXI: beige = normal, light beige = intermediate positive, light purple = intermediate negative, purple = deficient. Bold dashed lines represent the mean expression level of normal fibres. These data nicely confirm a loss of NDUFB8 subunit expression in many fibres, and to a lesser extent COX1 expression, consistent with a generalized defect in mitochondrial translation due to the *MT-TY* variant. (B) SDS-PAGE and one-dimensional BN-PAGE immunoblotting analysis of age-matched controls (C1, C2) and m.5860delTA *MT-TY* patient (P) mitochondrial membranes isolated from skeletal muscle showed decreased steady-state levels of Complex I subunits (NDUFB8 and MT-ND2) and mature Complex I. Complex II was used a loading control.

To determine the effects of the *MT-TY* m.5860delTA variant on mitochondrial function, quantitative fluorescent immunohistochemistry, western blotting and BN-PAGE analysis of key OXPHOS subunits and fully-assembled complexes were performed using patient skeletal muscle tissue. Quadruple immunofluorescence analysis confirmed a mitochondrial biochemical defect involving both complexes I and IV, with many fibres exhibiting a significant loss of NDUFB8 and COXI protein expression (Fig. 3**A**). In agreement with this result, immunoblot analysis of the steady-state levels of mitochondrial OXPHOS subunits showed a decrease in both the nuclear-encoded NDUFB8 and mitochondrially-encoded MT-ND2 subunit of Complex I (Fig. 3**B**). The protein levels of Complex III (UQCRC2), Complex IV (COX1) and Complex V (ATP5A) subunits were generally normal in the *MT-TY* patient compared to controls (Fig. 3**B**). Furthermore, subunits of Complex II (SDHA and SDHB) – a respiratory chain component that is entirely encoded by nuclear genes - were unaffected and served as loading controls.

The levels of fully assembled mitochondrial respiratory chain complexes were assessed by BN-PAGE and immunoblot analysis of mitochondrial extracts isolated from controls and *MT-TY* patient skeletal muscle tissue. Consistent with the decrease in steady-state levels of Complex I subunits (Fig. 3**B**), the m.5860delTA *MT-TY* variant also affected the assembly of Complex I showing a marked decrease in the amounts of fully assembled Complex I (Fig. 3**C**). The levels of Complexes II, III, IV and V in *MT-TY* patient's muscle were similar to controls (Fig. 3**C**). Taken together, these data suggest that the m.5860delTA variant preferentially affects the steady-state levels of Complex I components and their subsequent assembly into the mature OXPHOS holoenzyme.

## 4 Discussion

We describe the clinical, histochemical and molecular genetic findings in a paediatric patient due to a novel dinucleotide deletion in the *MT-TY* gene, where diagnostic muscle biopsy and full mitochondrial genome analysis was necessary to identify a mitochondrial aetiology. The case highlights the diagnostic challenge of mitochondrial disease at the bedside. Initially, clinical and neurophysiological features overlapped with a myasthenic syndrome, including exercise intolerance, proximal muscle weakness, ptosis, ophthalmoplegia and bulbar weakness and increased jitter on SFEMG. Although the finding of jitter from single fibre muscle electromyographic studies (SFEMG) suggested a myasthenic syndrome, previous studies in patients with mitochondrial myopathy and chronic progressive external ophthalmoplegia have also demonstrated slightly increased jitter [21], [22], [23]. Thus, in isolation, the presence of jitter on SFEMG is insufficient to distinguish myasthenia from mitochondrial myopathy.

The protracted diagnostic journey taken by this boy is not uncommon in neuromuscular disorders given that the myopathic symptoms which he had could overlap several neuromuscular diagnoses. In the absence of other features of mitochondrial disease (lactic acidosis, maternal pedigree of affected individuals and multisystem involvement), the neuromuscular features were initially regarded as a form of CMS. However, the lack of significant clinical response from pyridostigmine or salbutamol cast doubt on the diagnosis of CMS and consideration was then given to other conditions which might mimic CMS including congenital myopathies.

As is increasingly common in unsolved genetic neuromuscular disorders, the next line investigation was WES. This revealed two heterozygous *NEB* variants of unknown clinical significance. Nemaline myopathy due to mutations in *NEB* could have explained many of the clinical features of this case, including proximal and distal weakness and wasting, facial weakness, and respiratory muscle weakness, although ophthalmoplegia is unusual [24,25]. A subsequent muscle biopsy was performed with the aim of pursuing this genetic diagnosis. However, there were no rod-like structures on histological examination to support the diagnosis of nemaline myopathy. Instead, histochemical examination revealed a clear pattern of mosaic COX deficiency, providing a clue to the eventual diagnosis of mitochondrial myopathy.

Although mitochondrial disorders are characterised by a wide spectrum of clinical presentations, myopathy is a common finding [19,^26^,27]. The skeletal muscle biopsy has historically represented the ‘gold standard’ in the diagnostic work-up of mitochondrial disease because this post-mitotic tissue is densely packed with mitochondria, metabolically highly active, easily accessible and demonstrates histopathological correlates of disease. With decreasing sequencing costs and increasing quality, WES is commonly being used as a first-tier diagnostic tool for neuromuscular diseases, with invasive muscle biopsy being used as a complementary investigation if needed. However, as this case illustrates, in suspected mitochondrial diseases accurate interpretation of WES data may be compromised by a lack of prior knowledge of a respiratory chain defect and without the use of specific WES platforms which include mtDNA probes. In addition, any detected mtDNA variants of unknown clinical significance will require further functional validation in muscle in order to confirm their pathogenicity.

The muscle biopsy findings prompted full mtDNA analysis, leading to the genetic diagnosis of a novel m.5860delTA *MT-TY* variant, which we show fulfils canonical criteria to establish its pathogenicity. First, this variant is not listed on a large, publically-accessible mitochondrial genome database (http://www.mitomap.org/) which contains Genbank frequency data from 47,412 human mitochondrial DNA sequences nor was it present on our own in-house database of >1950 mtDNA sequences. Second, the highest level of the m.5860delTA variant was found in clinically-affected skeletal muscle (71%), with lower levels in blood (5%), buccal (4%) and urinary epithelial (28%) samples, exhibiting a tissue segregation that reflected disease manifestation. Third, this mutation appeared to have arisen *de novo* and was not detected in any DNA samples from his asymptomatic mother. Fourth, the m.5860delTA is located within the anticodon of the mt-tRNA^Tyr^ molecule leading to a generalised defect of mitochondrial translation, corroborated by our histochemical, immunohistochemical and protein studies (Fig. 3). Finally, single fibre segregation studies have established that the novel m.5860delTA *MT-TY* variant segregates with the COX histochemical defect in skeletal muscle, thus confirming its pathogenicity (Fig. 2**C**). Using a previously-validated scoring system [28] which includes numerous biochemical and molecular genetic parameters to assign pathogenicity to novel mt-tRNA variants, the m.5860delTA mutation easily exceeds the required threshold to distinguish itself from a neutral, polymorphic variant. This mutation is a deletion in the tRNA anti-codon acceptor of which has been listed in other mtDNA genes to be pathogenic [29].

The novel m.5860delTA variant is the first dinucleotide deletion to be reported in the *MT-TY* gene, and perhaps unsurprisingly given the functional consequences and severe biochemical defects, the onset of mitochondrial disease in childhood in our patient is one of the earliest amongst other reported cases, summarised in Table 1. Amongst all the reported clinical features, muscle involvement was the predominant phenotype, reflected by the high mutation load in this tissue. All other patients with pathogenic *MT-TY* variants reported proximal myopathy with the exception of one m.5843A>*G* carrier who presented with cardiomyopathy. Predominant bulbar weakness has been described in one other case, who had a pathogenic m.5835G>*A* variant.

**Table 1:** A summary of reported patients with pathogenic *MT-TY* gene variants.

***MT-TY* gene position**	m.5860delTA	m.5885delT	m.5877G>*A*	m.5874A>*G*	m.5843A>*G*	m.5835G>*A*	
**Number of cases**	1	1	1	1	1	2	
**Reference**	Current case	Raffelsberger et al. [1]	Sahashi et al. [2]	Pulkes et al. [3]	Scaglia et al. [4]	Kornblum et al. [5]	Simoncini et al. [6]
Sex	Male	Female	Female	Female	Female	Male	Female
Age of onset	4 years	35 years	28 years	5 years	3 years	15 years	51 years
**Clinical manifestations**							
Myopathy	Yes	Yes	Yes	Yes	No	No	Yes
Ophthalmoplegia	Yes	Yes	Yes	No	No	Yes	No
Ptosis	Yes	Yes	Yes	Yes	No	Yes	No
Exercise intolerance	Yes	No	No	Yes	No	Yes	No
Bulbar dysfunction	Yes	No	No	No	n/d	No	Yes
Other(s)	Learning difficulties	Myalgia post-exercise	Episodic diarrhoea	Non-specific stiffness in limbs	Focal segmental glomerulosclerosis, cardiomyopathy	High lactate post-exercise	Paravertebral and quadriceps hypotrophy
**Muscle biopsy findings**							
Respiratory chain enzyme deficiencies	Complex I & IV	Complex I & IV	n/d	Complex III & IV	Complex I	Complex I & IV	n/d
Red ragged fibres	>10%	Present	4%	24–73%	Not seen	Present	Present
COX-deficient fibres	>30%	Present	0.7%	24–73%	Not seen	Present	Present
**Heteroplasmy level**							
Buccal	4%	n/d	n/d	n/d	n/d	<5%	n/d
Blood	5%	<5%	0.7%	<5%	Homoplasmic	<5%	n/d
Urine	28%	n/d	n/d	n/d	n/d	n/d	n/d
Muscle	72%	78%	73%	89%	Homoplasmic	98%	95%

## 5 Conclusions

In summary, we report a novel m.5860delTA *MT-TY* gene variant presenting with childhood-onset mitochondrial myopathy characterised by ophthalmoplegia, ptosis, exercise intolerance, bulbar dysfunction and nocturnal hypoventilation. Extensive confirmatory functional work and supporting single-fibre segregation studies have proven the pathogenicity of this variant, illustrating the importance of a diagnostic muscle biopsy in guiding clinical-diagnostic pathways and allowing functional confirmation of rare genetic variants.

## References

[1] Schon E.A., DiMauro S., Hirano M (2012). Human mitochondrial DNA: roles of inherited and somatic mutations. Nat Rev Genet.

[2] Taylor R.W., Turnbull D.M. (2005). Mitochondrial DNA mutations in human disease. Nat Rev Genet.

[3] Yarham J.W., Elson J.L., Blakely E.L., McFarland R., Taylor R.W (2010). Mitochondrial tRNA mutations and disease. Wiley Interdiscip Rev RNA.

[4] Blakely E.L., Yarham J.W., Alston C.L., Craig K., Poulton J., Brierley C. (2013). Pathogenic mitochondrial tRNA point mutations: nine novel mutations affirm their importance as a cause of mitochondrial disease. Hum Mutat.

[5] Chaouch A., Porcelli V., Cox D., Edvardson S., Scarcia P., De Grassi A. (2014). Mutations in the Mitochondrial Citrate Carrier SLC25A1 are Associated with Impaired Neuromuscular Transmission. J Neuromuscul Dis.

[6] Guo Y., Menezes M.J., Menezes M.P., Liang J., Li D., Riley L.G. (2015). Delayed diagnosis of congenital myasthenia due to associated mitochondrial enzyme defect. Neuromuscul Disord.

[7] Plewnia K., Dotti M.T., Malandrini A., Manneschi L., Battisti C., De Stefano N. (1997). A rare association of myasthenia gravis and mitochondrial myopathy: a clinical, biochemical and morphologic study of one case. J Submicrosc Cytol Pathol.

[8] McFarland R., Elson J.L., Taylor R.W., Howell N., Turnbull D.M (2004). Assigning pathogenicity to mitochondrial tRNA mutations: when "definitely maybe" is not good enough. Trends Genet.

[9] Kornblum C., Zsurka G., Wiesner R.J., Schroder R., Kunz W.S (2008). Concerted action of two novel tRNA mtDNA point mutations in chronic progressive external ophthalmoplegia. Biosci Rep.

[10] Pulkes T., Siddiqui A., Morgan-Hughes J.A., Hanna M.G (2000). A novel mutation in the mitochondrial tRNA(Tyr) gene associated with exercise intolerance. Neurology.

[11] Raffelsberger T., Rossmanith W., Thaller-Antlanger H., Bittner R.E (2001). CPEO associated with a single nucleotide deletion in the mitochondrial tRNA(Tyr) gene. Neurology.

[12] Sahashi K., Yoneda M., Ohno K., Tanaka M., Ibi T., Sahashi K (2001). Functional characterisation of mitochondrial tRNA(Tyr)mutation (5877G>A) associated with familial chronic progressive external ophthalmoplegia. J Med Genet.

[13] Scaglia F., Vogel H., Hawkins E.P., Vladutiu G.D., Liu L-L, Wong L-JC (2003). Novel homoplasmic mutation in the mitochondrial tRNA(Tyr) gene associated with atypical mitochondrial cytopathy presenting with focal segmental glomerulosclerosis. Am J Med Genet Part A.

[14] Simoncini C., Montano V., Ali G., Costa R., Siciliano G., Mancuso M (2018). Proximal Myopathy due to m.5835G>A Mutation in Mitochondrial *MT-TY* Gene. Case Rep Neurol Med.

[15] Old S., Johnson M. (1989). Methods of microphotometric assay of succinate dehydrogenase and cytochrome c oxidase activities for use on human skeletal muscle. Histochem J.

[16] Rocha M.C., Grady J.P., Grunewald A., Vincent A., Dobson P.F., Taylor R.W. (2015). A novel immunofluorescent assay to investigate oxidative phosphorylation deficiency in mitochondrial myopathy: understanding mechanisms and improving diagnosis. Sci Rep.

[17] Andrews R.M., Kubacka I., Chinnery P.F., Lightowlers R.N., Turnbull D.M., Howell N (1999). Reanalysis and revision of the Cambridge reference sequence for human mitochondrial DNA. Nat Genet.

[18] Olahova M., Hardy S.A., Hall J., Yarham J.W., Haack T.B., Wilson W.C. (2015). LRPPRC mutations cause early-onset multisystem mitochondrial disease outside of the French-Canadian population. Brain.

[19] Kirby D.M., Thorburn D.R., Turnbull D.M., Taylor R.W (2007). Biochemical assays of respiratory chain complex activity. Methods Cell Biol..

[20] Blakely E.L., He L., Taylor R.W., Chinnery P.F., Lightowlers R.N., Schaefer A.M. (2004). Mitochondrial DNA deletion in "identical" twin brothers. J Med Genet.

[21] Krendel D.A., Sanders D.B., Massey J.M (1987). Single fiber electromyography in chronic progressive external ophthalmoplegia. Muscle Nerve: Off J Am Assoc Electrodiagnostic Med.

[22] Ukachoke C., Ashby P., Basinsk A., Sharpe J (1994). Usefulness of single fiber EMG for distinguishing neuromuscular from other causes of ocular muscle weakness. Can J Neurol Sci.

[23] Braz L.P., Ng Y.S., Gorman G.S., Schaefer A.M., McFarland R., Taylor R.W., et al. Neuromuscular junction abnormalities in mitochondrial disease. An observational cohort study. 2019:10.1212/CPJ.0000000000000795. 10.1212/CPJ.0000000000000795 PMC8032443 33842062

[24] Sewry C.A., Laitila J.M., Wallgren-Pettersson C (2019). Nemaline myopathies: a current view. J Muscle Res Cell Motil.

[25] Lehtokari V.L., Kiiski K., Sandaradura S.A., Laporte J., Repo P., Frey J.A. (2014). Mutation update: the spectra of nebulin variants and associated myopathies. Hum Mutat.

[26] Taylor R.W., Schaefer A.M., Barron M.J., McFarland R., Turnbull D.M (2004). The diagnosis of mitochondrial muscle disease. Neuromuscul Disord.

[27] Alston C.L., Rocha M.C., Lax N.Z., Turnbull D.M., Taylor R.W (2017). The genetics and pathology of mitochondrial disease. J Pathol.

[28] Yarham J.W., Al-Dosary M., Blakely E.L., Alston C.L., Taylor R.W., Elson J.L. (2011). A comparative analysis approach to determining the pathogenicity of mitochondrial tRNA mutations. Hum Mutat.

[29] Joshi P.R., Baty K., Hopton S., Cordts I., Falkous G., Schoser B. (2020). Progressive external ophthalmoplegia due to a recurrent de novo m.15990C>T MT-TP (mt-tRNA(Pro)) gene variant. Neuromuscul Disord.

